# Development and validation of a questionnaire to evaluate the knowledge, attitude and practices regarding travel medicine amongst physicians in an apex tertiary hospital in Northern India

**DOI:** 10.1186/s40794-022-00170-w

**Published:** 2022-06-01

**Authors:** Arvind Kumar, Anand Rajendran, Mohd Usman, Jatin Ahuja, Sameer Samad, Ankit Mittal, Prerna Garg, Upendra Baitha, Piyush Ranjan, Naveet Wig

**Affiliations:** 1 Department of Medicine, AIIMS, New Delhi, India; 2 Infectious Diseases & Travel Health Specialist, Indraprastha Apollo Hospital, New Delhi, India

**Keywords:** Travel Medicine, Knowledge, Practice, Questionnaire, Development, Validation

## Abstract

**Objectives:**

Travel medicine focuses primarily on pre-travel preventive care and the conditions and diseases acquired during or after travel. There is a paucity of validated tools to assess the knowledge, attitude and practises of physicians with regard to travel medicine. We attempted to develop a tool to assess existing expertise among Medicine and Infectious Diseases resident doctors with respect to travel medicine.

**Methods:**

Item level content validity index (I-CVI) and scale level content validity index (S-CVI/Ave) were estimated for each of the items to establish the content validity. Refined measures of inter-rater agreement (Brennan and Prediger Agreement Coefficient and Gwet’s Agreement Coefficient) were estimated for the tool.

**Results:**

The final version of the questionnaire had satisfactory content validity (I-CVI > 0∙6 and S-CVI/Ave > 0∙9) and possessed high agreement among the raters (Brennan and Prediger AC > 0∙7, *p* < 0∙01 and Gwet's AC > 0∙8, *p* < 0∙01) with regard to necessity, clarity and relevance of the scale.

**Conclusions:**

This tool covers a wide range of questions and is scientifically validated. The final version of the tool can be used largely for the assessment of knowledge, attitude and practices among medical practitioners. This is instrumental to build targeted intervention programs to enhance the knowledge regarding travel medicine among health care providers.

**Supplementary Information:**

The online version contains supplementary material available at 10.1186/s40794-022-00170-w.

## Introduction

After a long gap of the Spanish flu pandemic, the emergence of COVID-19 has taught many lessons to humanity. One of the key message is to realize the importance of public health from a global perspective. The health care system should be geographically inclusive and not be confined to a region or a country because a pandemic like this has no boundaries. Travel medicine globalizes health care in terms of providing preventive and curative health across boundaries.

Travel medicine or emporiatrics is the field of medicine which is concerned with the promotion and protection of health of travellers. It aims to prevent diseases and other adverse health outcomes among international travellers. It requires up-to-date information on the global epidemiology of the non-infectious and infectious health risks, health regulations and vaccination necessities in various countries along with the emerging patterns of medication-resistant infections [1]. As international travel becomes more accessible, knowledge of this field is likely to become essential for a physician [2, 3]. Although derived from the traditional medicine disciplines, this branch of medicine is a newly emerging field given the increasing number of international travellers and reporting of various infectious and non-infectious diseases [4], injuries [5] and other health risks among international travellers [6–8]. Since travel medicine is a new discipline, expert opinion and experience still dominate many areas in this branch, highlighting the need for continuous investigation in the field [9].

International travellers are at higher risk of developing various health threats, which depend on both the health needs of the traveller and on the type of travel to be undertaken. The traveller’s triad includes the three major components that influence the risk associated with a specific travel plan i.e. place, time and person. The region of the world being visited determines the altitude, humidity, temperature and infection profile etc. The travellers’ vulnerability to these exposures may be determined by their age, general well-being, the trip's length, and the diversity of planned activities [10]. Pre-travel health education, vaccination and prophylactic drugs may serve to mitigate these risks [10–12].

With rapidly evolving travel regulations, there is a need to provide training to practicing physicians to predict travel-associated health risks and recognise untoward exposures. As travel medicine gains prominence worldwide, we recognise the dearth of adequately trained field experts. In the absence of subject specialists, general physicians must be provided formal training to ensure adequate care [13]. In this regard, an assessment of the existing knowledge among health care practitioners is necessary to develop interventions for targeting gaps in knowledge.

With this objective in mind, an attempt was made at our tertiary care facility to develop a comprehensive tool covering major aspects of travel medicine. Currently, no widely disseminated, valid instrument for assessing travel medicine's knowledge, attitude, and practices is available in India. This tool assesses the knowledge, attitude and practices (KAP) regarding travel medicine in the form of a questionnaire. We also attempted to validate this tool in primary care physicians, internist, infectious disease specialists, and other health care providers.

## Material and methods

### Development and validation of the travel medicine questionnaire

#### Step I: Conceptualization and identification of domains and sub-domains for the travel medicine knowledge, attitude and practices assessment tool

**Fig. 1:**
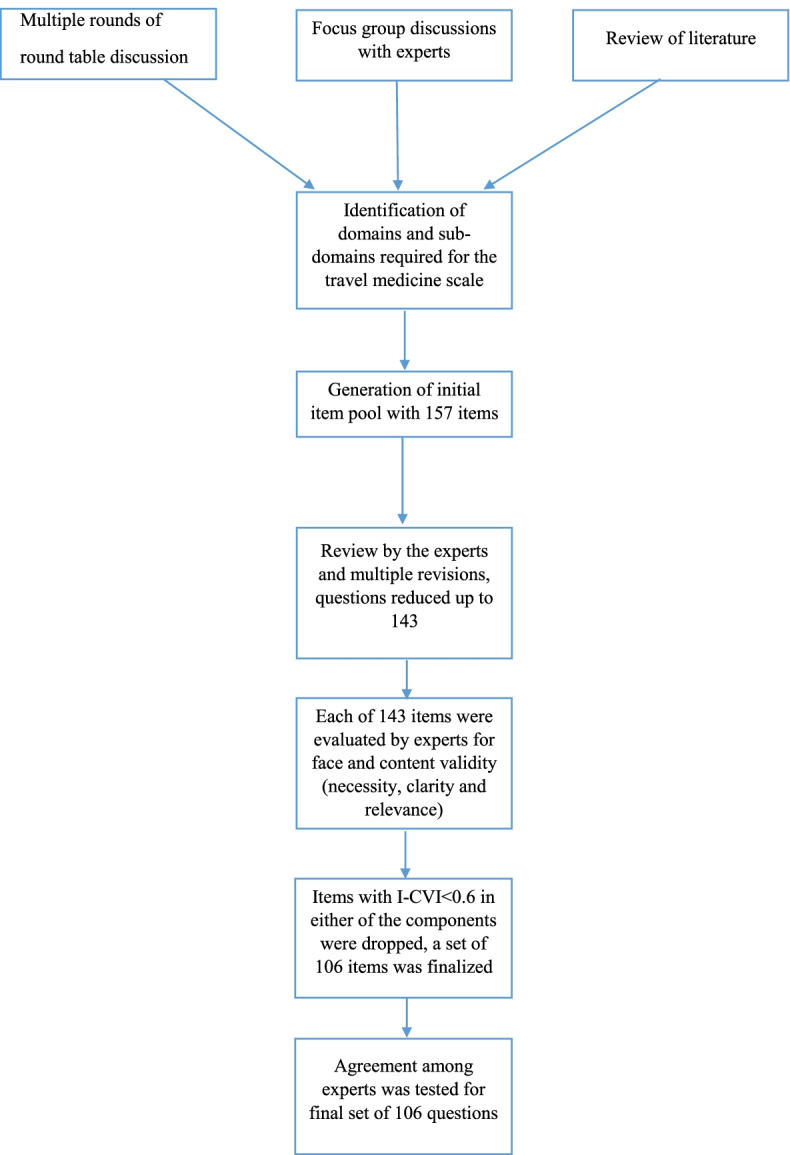
Flow chart of the development and validity of the travel medicine tool

For conceptualizing and identification of domains and sub-domains to develop the initial pool of items, multiple round table discussions and focus group discussions (FGDs) were held among experts from different fields of medicine, infectious diseases and travel medicine from five tertiary care centers in different parts of India. This included subject experts with certifications in travel medicine from the International Society of Travel Medicine (ISTM) and physicians currently practicing in travel clinics across the country (Fig. 1).

#### Step II: Literature review to identify domains and sub-domains and generating preliminary item pool for the tool

An extensive literature review was carried out to analyse the existing evidence on travel medicine. The literature search aimed to identify domains and sub-domains required to develop the travel medicine KAP assessment tool. We used the standard textbooks, journals, and internet databases for the identification of relevant concepts. The internet search engines used were Google Scholar, PubMed, Scopus and JSTOR using the keywords string: (Travel Medicine) AND (Knowledge) AND (Attitude) AND (Practices) published in or after year 2000. Table 1 shows the details of some of the sources used to identify the domains and sub-domains and to generate a preliminary item pool for our travel medicine tool.

**Table 1:** List of various books/journals used to identify the domains and sub-domains and to develop the preliminary pool of items for travel medicine tool

Name of the Journal/Book	Name of the author(s)/editor(s)	Name of the Publisher	Publication year/reference period
1.Principles and Practice of Travel Medicine	Jane N. Zuckerman	John Wiley & Sons	2013
2.Travel Medicine	Jay S. Keystone Phyllis E. Kozarsky Bradley A. Connor Hans D. Nothdurft Marc Mendelson Karin Leder	Elsevier	2019
3.Manual of Travel Medicine	Joseph Torresi Sarah McGuinness Karin Leder Daniel O'Brien Tilman Ruff Mike Starr Katherine Gibney	Springer	2019
4.CDC Health Information for International Travel 2018. The Yellow Book	Gary W. Brunette	Oxford University Press	2018
5.Manual of Travel Medicine and Health	Robert Steffen Herbert L. DuPont Annelies Wilder-Smith	BC Decker Inc	2003
6.Journal of Travel Medicine	Annelies Wilder-Smith	Oxford University Press	2000 or later
7.Travel Medicine and Infectious Disease	Patricia Schlagenhauf-Lawlor	Elsevier	2000 or later
8.International Travel and Health	Gilles Poumerol Annelies Wilder-Smith	World Health Organization (WHO)	2012

#### Step-III: Developing the structure of the questionnaire through expert review

The initial pool of 157 questions was again reviewed by the experts, and the number of items was reduced to 143. This set of identified items was organized into the form of a questionnaire. The questionnaire was constructed in a simple and lucid language, and the flow of the questions was maintained, keeping in mind the purpose of assessment of KAP.

#### Step-IV: Establishing face and content validity and estimation of agreement coefficients

Face validity is the lowest level of validity and represents the assumption of an expert and acceptance that a test represents the domain being assessed. After preparing the first draft, the questionnaire was reviewed by the experts in the field for face validity (by assessing for readability, comprehensibility, feasibility, completeness and layout and style).

The purpose of content validation is to reduce the bias associated with the operationalization of the instrument in the initial stages [14]. To establish the content validity of the travel medicine tool, we chose three components to judge the overall content validity. Studies estimate the content validity indices with a single validity parameter. However, a few studies adopted a different approach and decomposed the overall validity into its components [15, 16]. We adopted this technique and judged the overall content validity in terms of—Necessity (Is the question necessary to be asked to the resident to assess their knowledge/attitude/practices of travel medicine?), Clarity (Is the question wording/structure/options given convey the meaning effectively?) and Relevance (Is the question relevant as far as branch of travel medicine is concerned with respect to knowledge/attitude/practice?). Each question was evaluated by the experts for these components separately. The rating protocol was designed into the form of a Likert scale as-

- 1) Necessity (N): Each item was rated as: 1 (neither useful nor necessary), 2 (useful but not necessary) and 3 (essential).
- 2) Clarity (C): Rate each item as 1(not clear), 2(slightly clear/needs major revision), 3 (clear/needs minor revision), 4 (very clear).
- 3) Relevance (R): Each item was rated as: 1 (not relevant), 2 (slightly relevant/needs major revision), 3 (relevant/needs minor revision), 4 (very relevant).

The idea behind decomposing the overall quality judgement into its components (N, C and R) was to give more freedom to the experts to judge and to provide more strength to the validation process. Furthermore, we also looked for any lack of consistency between experts for travel medicine questionnaire in terms of three above mentioned parameters.

### Measures

We chose several measures of inter-rater agreement as well as indices of validity to validate the travel medicine tool.

#### Content validity indices (CVI)

Two indices have been proposed by researchers for judgement of content validity of a tool. This includes-item level content validity index (I-CVI) and scale level content validity index (S-CVI)[17]. The eight experts rated each of the item in terms of N, C and R as mentioned above. In the next step, these scores were dichotomized. For necessity, the dichotomous variable was categorized as ‘1’ if the item was rated as ‘3’ (essential) or ‘0’ otherwise. Likewise, for clarity and relevance the dichotomous variables were generated as ‘1’ for experts giving a rating of 3 or 4 and ‘0’ otherwise. These dichotomous variables were then used to estimate the content validity indices (I-CVI and S-CVI/Ave) for each of these characteristics. The item-level CVI (I-CVI) is computed by dividing the total number of ‘1’s by the total number of experts [17, 18]. The S-CVI/Ave is then calculated by averaging the I-CVIs estimated for each item of the instrument. This exercise was repeated for each of the parameters of content validity (necessity, clarity and relevance). Polit and Beck [17] recommended an S-CVI/Ave of 0∙90 or above as excellent.

#### Agreement Coefficients (AC)

The most popular method of quantification of inter-rater agreement among researchers has been the Cohen’s Kappa [19–22]. Recent published literature discussed the limitations of kappa statistic and proposed other measures of inter-rater agreement [19, 22]. Klein [19] has pointed out the limitations of Cohen’s Kappa and suggested that the Brennan and Prediger [23] coefficient and Gwet’s [24, 25] agreement coefficient arguably represent the data more accurately. Further, he suggested that these two agreement measures are found to be more robust than any other measure of inter rater agreement [19]. Similarly, Wongpakaran [22] found Gwet’s AC to provide a more stable inter-rater reliability coefficient than Cohen’s Kappa and recommended to use for inter-rater reliability analysis. A more detailed discussion on each of the agreement coefficients may be found elsewhere [19, 22, 25]. In our study, we estimated these agreement coefficients along with the percent agreement for each of the three above mentioned components.

## Results

### Content validity indices

Table 1 shows the I-CVIs and S-CVI/Ave for each of the component of content validity. Polit and Beck recommended an I-CVI ≥ 0∙78 for inclusion of an item [17]. But, we adopted a less strict cut-off of I-CVI < 0∙60 for deletion of the items from the pool [26]. We removed the items which had an I-CVI < 0∙6. The I-CVIs for necessity ranged from 0∙625 to 1∙000. Six items had a necessity I-CVI of 0∙625 whereas five items had 0∙750. Rest of the items had an I-CVI ≥ 0∙78 where out of total 106 items 85 items had an I-CVI of 1∙000. This reflects satisfactory ranges for necessity in terms of I-CVIs. For clarity, we observed a slightly better I-CVIs which ranged from 0∙875 to 1∙000 with six items having an I-CVI of 0∙875 and rest of the items had 1∙000. Likewise, for relevance the I-CVIs ranged from 0∙625 to 1∙000 where four items had an I-CVI of 0∙625. The overall scale level content validity index (S-CVI/Ave) was observed to be above 0∙900 for each of the dimension of content validation.

### Agreement among the experts

The final version of questionnaire had 106 items after removal of items with an I-CVI < 0∙6 (Supplementary Table 1). For the remaining items, we estimated agreement indicators. The results of these agreement indicators with regard to the three dimensions of overall quality of the tool has been depicted in Table 2. We observed a high and statistically significant percentage agreement among experts with regard to the overall validity of the travel medicine tool. For each of the dimensions, the overall percent agreement among the experts was above 90 percent. The cut-offs of agreement according to Gwet’s AC as categorized by Tammaa [27] is as follows: < 0∙2 = poor; 0∙21–0∙4 = fair; 0∙41–0∙6 = moderate; 0∙61–0∙8 = substantial; and 0∙81–1∙0 = almost perfect. For each of the dimensions, we observed Gwet’s AC > 0∙8 showing high levels of agreement among raters (Table 3).

**Table 2:** Item level content validity index (I-CVI) for each item and scale level content validity index for travel medicine questionnaire

	I-CVI				I-CVI				I-CVI		
Item no∙	Necessity (N)	Clarity (C)	Relevance (R)	Item no∙	Necessity (N)	Clarity (C)	Relevance (R)	Item no∙	Necessity (N)	Clarity (C)	Relevance (R)
1	1∙000	1∙000	1∙000	41	1∙000	1∙000	1∙000	81	1∙000	1∙000	0∙875
2	1∙000	1∙000	1∙000	42	1∙000	1∙000	1∙000	82	1∙000	0∙875	1∙000
3	0∙625	1∙000	0∙625	43	1∙000	1∙000	1∙000	83	0∙875	0∙875	1∙000
4	0∙875	1∙000	1∙000	44	1∙000	1∙000	1∙000	84	1∙000	1∙000	1∙000
5	1∙000	1∙000	1∙000	45	1∙000	1∙000	1∙000	85	1∙000	1∙000	1∙000
6	1∙000	1∙000	1∙000	46	1∙000	1∙000	1∙000	86	1∙000	1∙000	1∙000
7	1∙000	1∙000	1∙000	47	0∙625	1∙000	0∙625	87	1∙000	1∙000	1∙000
8	0∙875	1∙000	1∙000	48	1∙000	1∙000	1∙000	88	1∙000	1∙000	1∙000
9	1∙000	1∙000	1∙000	49	1∙000	1∙000	1∙000	89	1∙000	1∙000	1∙000
10	1∙000	1∙000	1∙000	50	0∙625	0∙875	0∙750	90	1∙000	1∙000	1∙000
11	1∙000	1∙000	1∙000	51	0∙875	0∙875	1∙000	91	0∙750	1∙000	0∙625
12	1∙000	1∙000	1∙000	52	0∙875	1∙000	1∙000	92	1∙000	1∙000	1∙000
13	1∙000	1∙000	1∙000	53	1∙000	1∙000	1∙000	93	1∙000	1∙000	1∙000
14	1∙000	1∙000	1∙000	54	1∙000	1∙000	1∙000	94	1∙000	1∙000	1∙000
15	1∙000	1∙000	1∙000	55	0∙875	1∙000	0∙875	95	1∙000	1∙000	1∙000
16	1∙000	1∙000	1∙000	56	1∙000	1∙000	1∙000	96	1∙000	1∙000	1∙000
17	0∙625	1∙000	0∙875	57	1∙000	1∙000	1∙000	97	1∙000	1∙000	1∙000
18	1∙000	1∙000	1∙000	58	1∙000	1∙000	1∙000	98	1∙000	1∙000	1∙000
19	1∙000	1∙000	1∙000	59	1∙000	1∙000	1∙000	99	1∙000	1∙000	1∙000
20	1∙000	1∙000	1∙000	60	1∙000	1∙000	1∙000	100	1∙000	1∙000	1∙000
21	1∙000	1∙000	1∙000	61	1∙000	1∙000	1∙000	101	1∙000	1∙000	1∙000
22	1∙000	1∙000	1∙000	62	1∙000	1∙000	1∙000	102	1∙000	1∙000	1∙000
23	1∙000	1∙000	1∙000	63	1∙000	1∙000	1∙000	103	1∙000	1∙000	1∙000
24	0∙875	1∙000	1∙000	64	1∙000	1∙000	1∙000	104	1∙000	1∙000	1∙000
25	0∙875	1∙000	0∙875	65	1∙000	0∙875	1∙000	105	1∙000	1∙000	1∙000
26	0∙750	1∙000	0∙625	66	1∙000	1∙000	1∙000	106	1∙000	1∙000	1∙000
27	0∙750	1∙000	0∙750	67	1∙000	1∙000	1∙000	**S-CVI/Ave**	**0∙955**	**0∙994**	**0∙968**
28	1∙000	1∙000	1∙000	68	1∙000	1∙000	1∙000				
29	1∙000	1∙000	1∙000	69	1∙000	1∙000	1∙000				
30	1∙000	1∙000	1∙000	70	0∙750	1∙000	0∙875				
31	1∙000	1∙000	1∙000	71	1∙000	1∙000	1∙000				
32	1∙000	1∙000	1∙000	72	1∙000	1∙000	1∙000				
33	0∙875	1∙000	1∙000	73	0∙625	1∙000	0∙750				
34	1∙000	1∙000	1∙000	74	0∙875	1∙000	0∙750				
35	1∙000	1∙000	1∙000	75	0∙750	1∙000	1∙000				
36	1∙000	1∙000	1∙000	76	1∙000	1∙000	1∙000				
37	1∙000	1∙000	1∙000	77	0∙875	0∙875	0∙875				
38	1∙000	1∙000	1∙000	78	1∙000	1∙000	1∙000				
39	0∙625	1∙000	0∙875	79	1∙000	1∙000	1∙000				
40	1∙000	1∙000	1∙000	80	1∙000	1∙000	1∙000

**Table 3:** Details of agreement among experts for travel medicine tool with regard to necessity, clarity and relevance

Indicator	Necessity	Clarity	Relevance
% Agreement	96∙8***	91∙4***	90∙8***
Brennan and Prediger AC	0∙9148***	0∙7258***	0∙7042***
Gwet's AC	0∙9656***	0∙8445***	0∙8336***

^***^p < *0∙01*

## Discussion

With the increasing number of international travellers, travel medicine has gained new significance. Studies have highlighted that the prevalence of travel related problems is surprisingly high among the travellers [7, 28–30] and also pointed out the inadequacies in KAP in both health providers as well as the travellers [31–34]. In order to build any specific intervention program to increase knowledge regarding travel medicine in healthcare practitioners, it becomes necessary to assess the existing knowledge of the health providers.

Earlier KAP studies in travel medicine are either disease-specific [31, 35] or conducted amongst travellers [32, 33, 35, 36]. None of these studies have elucidated on the development process of their tools, and validation data on the same is often missing. Very few studies have been published which attempted to develop and validate the questionnaire regarding travel medicine among medical practitioners. Ratnam et.al. developed and validated a questionnaire to assess the risk of developing viral infections in Australian Travellers [37]. The study covers only a particular domain (viral infections) of travel related problems among travellers and does not establish the content validity through estimation of content validity indices (I-CVI and S-CVI).

The major strength of this study is the development and validation of a travel medicine tool, which will enable the researchers to assess the KAP among health care providers. The content collection through thorough literature review as well as several rounds of discussion with the experts ensured the quality and coverage. Further, the establishment of content validity through expert evaluation and measures of content validity and agreement coefficients made the tool robust and scientifically validated. The final set of 106 questions had satisfactory content validity indices (I-CVI > 0∙6 and S-CVI/Ave > 0∙9). The agreement coefficients (Brennan and Prediger AC > 0∙7, *p* < 0∙01 and Gwet's AC > 0∙8, *p* < 0∙01) among the raters with regard to necessity, clarity and relevance of the travel medicine KAP assessment tool were observed to be high.

This study is not free from limitations. The experts chosen for reviewing the travel medicine tool are from internal medicine, infectious disease programme and allied branches who are involved in operating clinics of travel medicine since dedicated travel medicine branch is yet to evolve in India. One who has completed a certificate course in Travel Health from International Society of Travel Medicine (ISTM) was actively involve as an expert. Although, we have taken utmost care to cover every aspect of travel medicine, since it is a vast discipline there is always a prospect of modification and improvement of this tool. Due to limited resources available for the study, only experts from India were involved to review the travel medicine KAP tool. However, AIIMS, New Delhi being an apex health care center of India has specialists from all clinical domains of human health. Therefore, their expertise was used for the development of initial pool of items and revised version of the tool.

The patterns of infectious diseases vary by geographic region and population [38] and differences in the climate of various regions also impact the patterns of infectious diseases [39] and therefore require special attention by health care providers. We suggest that the definition of travel medicine should be expanded in such a way that it covers the health problems of domestic travellers and repatriates, to prevent the spread of infectious diseases especially various kinds of respiratory tract infections (RTIs) which may be highly contagious and can give rise to a pandemic. So, comprehensive attempts should be made to make the definition more exhaustive and the possible inclusion of this aspect should be the point of consideration in future.

## Conclusions

The pre-travel consultation has become a necessary part of the travellers’ checklist. Considering this issue, present study is a significant contribution in the field of travel medicine and provides the basis for the assessment of the knowledge, attitude and practices among medical practitioners so that adequate intervention programs may be developed to enhance the knowledge of travel medicine among health care providers. This tool covers a wide range of questions and is scientifically validated. The final version of the tool can be used globally for the assessment of knowledge, attitude and practices among medical practitioners. This is instrumental to build targeted intervention programs to enhance the knowledge regarding travel medicine among health care providers.

## Supplementary Information

**Additional file 1.** Questionnaire toEvaluate the Knowledge, Attitude and Practices Regarding Travel MedicineAmongst Physicians.

## Data Availability

The dataset supporting the conclusions of this article is available by taking prior approval of the corresponding author.
