# Supplementary material for: Development and validation of a questionnaire to evaluate the knowledge, attitude and practices regarding travel medicine amongst physicians in an apex tertiary hospital in Northern India
Source: Trop Dis Travel Med Vaccines. 2022 Jun 1;8:13. doi: 10.1186/s40794-022-00170-w (PMC9155205; doi:10.1186/s40794-022-00170-w)
Supplement: Supplementary file 1 — Additional file 1. Questionnaire toEvaluate the Knowledge, Attitude and Practices Regarding Travel MedicineAmongst Physicians. [file 40794_2022_170_MOESM1_ESM.docx]

**Additional file 1:** **Questionnaire to Evaluate the Knowledge, Attitude and Practices Regarding Travel Medicine Amongst Physicians**

| **Knowledge** | |
| --- | --- |
| 1 | Are you aware that there is mortality and morbidity specifically associated with travel? (0=no;1= not sure; 2=yes) |
| 2 | Are you aware of the existence of a branch of medicine called travel medicine? (0=no;1= not sure; 2=yes) |
| 3 | Are you aware of all three components of travel triad? (0=no;1= not sure; 2=yes) |
| 4 | Are you aware of the region wise prevalence of infectious disease so that you can give travel related health advice (regarding infection prevention) to a person visiting your country? (rate your knowledge from 0 to 4 ,0=not aware at all ;4 = very well aware) |
| 5 | Do you know the components in a basic travel medical kit? (0=no;1=not sure; 2=yes) |
| 6 | Are you aware of the factors to be considered in a pre-travel assessment? (rate your knowledge from 0 to 4 ,0=not aware at all ;4 = very well aware) |
| 7 | How much confident are you in foreseeing travel related health risk from the itinerary (rate your confidence from 0 to 4 ,0=not confident at all,4=very much confident) |
| 8 | Are you aware of the risk factors for motion sickness? (rate your knowledge from 0 to 4 ,0=not aware at all ;4 = very well aware) |
| 9 | Which among the following drugs has not shown to be effective in the management of motion sickness? (1=promethazine;2=prochlorperazine;3=hyoscine;4=ondansetron; 5=metoclopramide) |
| 10 | Are you aware of the absolute contraindications for long distance travel in case of a pregnant woman? (rate your knowledge from 0 to 4 ,0=not aware at all ;4 = very well aware) |
| 11 | Are you aware of the special components in pre-travel assessment of infants and small children (rate your knowledge from 0 to 4 ,0=not aware at all ;4 = very well aware) |
| 12 | Are you aware of the pre-travel considerations in case of travellers with pre-existing cardiovascular disease? (rate your knowledge from 0 to 4 ,0=not aware at all ;4 = very well aware) |
| 13 | Are you aware of the health-related contraindications of air travel? (rate your knowledge from 0 to 4 ,0=not aware at all ;4 = very well aware) |
| 14 | Are you aware of the specific health related issues that can arise as a result of prolonged air travel? (rate your knowledge from 0 to 4 ,0=not aware at all ;4 = very well aware) |
| 15 | Are you aware of the treatment options for jet lag? (rate your knowledge from 0 to 4 ,0=not aware at all ;4 = very well aware) |
| 16 | Are you aware of the measures to avoid development of Deep Vein Thrombosis due to air travel? (rate your knowledge from 0 to 4 ,0=not aware at all ;4 = very well aware) |
| 17 | Are you aware the practical advices that can be given to a wheelchair traveller before air travel? (rate your knowledge from 0 to 4 ,0=not aware at all ;4 = very well aware) |
| 18 | Are you aware of the measures to be taken when a passenger with ear/nose/throat infection prepares for air travel? (rate your knowledge from 0 to 4 ,0=not aware at all ;4 = very well aware) |
| 19 | Are you aware of the additional health risks during travel by sea? (rate your knowledge from 0 to 4 ,0=not aware at all ;4 = very well aware) |
| 20 | Are you familiar with the format and rules regarding international certificate of vaccination or prophylaxis? (rate from 0 to 4, 0=not familiar at all;4=very much familiar) |
| 21 | Are you aware of the guidelines for vaccination of travellers? (rate your knowledge from 0 to 4 ,0=not aware at all ;4 = very well aware) |
| 22 | Are you aware of the indications for Hepatitis A vaccination? (rate your knowledge from 0 to 4 ,0=not aware at all ;4 = very well aware) |
| 23 | Are you aware of the indications for yellow fever vaccination? (rate your knowledge from 0 to 4 ,0=not aware at all ;4 = very well aware) |
| 24 | Are you aware of the contraindications of yellow fever vaccination (rate your knowledge from 0 to 4 ,0=not aware at all ;4 = very well aware) |
| 25 | Will you be able to enlist the nearby centres where yellow fever vaccine can be administered to your patient? (rate your ability from 0 to 4 ,0=very unlikely ;4 = very likely) |
| 26 | Are you aware of the regulations regarding waiver letter for not receiving YF vaccination? (rate your knowledge from 0 to 4 ,0=not aware at all ;4 = very well aware) |
| 27 | Will you be able to tell your patient when simultaneous administration of 2 or more vaccines are contraindicated? (0=no;1= not sure; 2=yes) |
| 28 | Will you be able to prescribe correctly the schedule of HBV vaccine to a previously unimmunised adult traveller? (rate from 0 to 4 ,0=very unlikely ;4 = very likely) |
| 29 | Are you aware of the indications for administering typhoid vaccination (Vi polysaccharide) before travel? (rate your knowledge from 0 to 4 ,0=not aware at all ;4 = very well aware) |
| 30 | Are you aware of the indications of Japanese Encephalitis (JE) vaccine (rate your knowledge from 0 to 4 ,0=not aware at all ;4 = very well aware) |
| 31 | Are you aware of the duration of immunity offered by single dose of JE vaccine? (rate your knowledge from 0 to 4 ,0=not aware at all ;4 = very well aware) |
| 32 | Are you aware of the classic meningitis prone area, travel to which requires meningococcal vaccine? (rate your knowledge from 0 to 4 ,0=not aware at all ;4 = very well aware) |
| 33 | Do you know the vaccination requirements for visiting USA as per the CDC guidelines? (rate your knowledge from 0 to 4 ,0=not aware at all ;4 = very well aware) |
| 34 | Will you be able to counsel about the need for vaccination to a traveller/patient who is not willing for vaccination? (rate your ability from 0 to 4, 0=very unlikely, 4=very likely) |
| 35 | Are you aware of the contraindications for travel to high altitude? (rate your knowledge from 0 to 4 ,0=not aware at all ;4 = very well aware) |
| 36 | Are you aware of the precautions to be taken by travellers (unaccustomed to high altitude) before travelling to high altitude? (rate your knowledge from 0 to 4 ,0=not aware at all ;4 = very well aware) |
| 37 | Are you aware of the precipitating factors for high altitude illness? (rate your knowledge from 0 to 4, 0=not aware at all ;4 = very well aware) |
| 38 | Will you be able to explain the symptoms of acute mountain sickness to a patient planning for travel to high altitude? (rate from 0 to 4 ,0=very unlikely ;4 = very likely) |
| 39 | Are you aware of the management approach for high altitude illness? (rate your knowledge from 0 to 4 ,0=not aware at all ;4 = very well aware) |
| 40 | Are you aware of the use of portable hyperbaric chamber for the management of high-altitude illness? (rate your knowledge from 0 to 4 ,0=not aware at all ;4 = very well aware) |
| 41 | Will you be able to instruct the use of acetazolamide for the prophylaxis of mountain sickness? (rate from 0 to 4 ,0=very unlikely ;4 = very likely) |
| 42 | Are you aware of the health risk associated with hot climate? (rate your knowledge from 0 to 4 ,0=not aware at all ;4 = very well aware) |
| 43 | Are you familiar with the management guidelines for heat stroke? (rate your knowledge from 0 to 4, 0=not at all familiar; 4=very much familiar) |
| 44 | Are you aware of the health risks associated with cold climate? (rate your knowledge from 0 to 4 ,0=not aware at all ;4 = very well aware) |
| 45 | Are you familiar with the management guidelines for frostbite? (rate your knowledge from 0 to 4, 0=not at all familiar; 4=very much familiar) |
| 46 | Are you aware of the issues to be addressed when your patient is planning for deep sea diving? (rate your knowledge from 0 to 4 ,0=not aware at all ;4 = very well aware) |
| 47 | Are you aware of the absolute contraindications for scuba diving by recreational scuba training council? (rate your knowledge from 0 to 4 ,0=not aware at all ;4 = very well aware) |
| 48 | Are you familiar with the management guidelines for decompression sickness? (rate you knowledge from 0 to 4, 0=not at all familiar;4=very much familiar) |
| 49 | Are you aware of the specific considerations before wilderness travel? (rate your knowledge from 0 to 4 ,0=not aware at all ;4 = very well aware) |
| 50 | Are you aware of the specific health related challenges associated with mass gatherings? (rate your knowledge from 0 to 4 ,0=not aware at all ;4 = very well aware) |
| 51 | Will you be able to prescribe vaccinations for a traveller preparing for Hajj pilgrimage? (rate your ability from 0 to 4, 0=very unlikely;4=very likely) |
| 52 | Will you be able to explain the different methods of personal protection against insect bite to your patient? (rate your ability from 0 to 4, 0=very unlikely ;4=very likely) |
| 53 | Will you be able to explain the safe and effective use of insect repellent to your patient? (rate from 0 to 4, 0=very unlikely ;4=very likely) |
| 54 | Are you familiar with the post and pre-exposure prophylaxis against rabies? ( rate from 0 to 4, 0=not at all familiar;4=very much familiar) |
| 55 | Will you be able to advise the precautions to be taken to avoid health hazards associated with recreational water to your patient (rate your ability from 0 to 4, 0=very unlikely, 4=very likely) |

| 56 | Will you be able to explain the situations where one should avoid swimming? (rate your ability from 0 to 4, 0=very unlikely, 4=very likely) |
| --- | --- |
| 57 | Are you confident enough to give advice regarding the practical measures for food and water hygiene? (rate between 0 to 4, 0=not at all confident;4=very much confident) |
| 58 | Will you be able to explain any technique for field water disinfection for a traveller planning for travel to a remote area? (rate your ability from 0 to 4, 0=very unlikely, 4=very likely) |
| 59 | Are you aware of the recommendations regarding practicing of sexual activity (safe sexual practices) before, during and after travel? (rate your knowledge from 0 to 4 ,0=not at all aware;4=very well aware) |
| 60 | Will you be able to prescribe HIV Post Exposure Prophylaxis for a patient eligible for it? (rate your ability from 0 to 4, 0=very unlikely, 4=very likely) |
| 61 | Are you aware of the indications and regimen for HIV Pre exposure prophylaxis? (rate your knowledge from 0 to 4 ,0=not at all aware;4=very well aware) |
| 62 | Are you aware about the groups of people who are at particular risk of severe malaria? (rate your knowledge from 0 to 4 ,0=not at all aware;4=very well aware) |
| 63 | Are you aware of the guidelines for Stand by Emergency Treatment for Malaria? (rate your knowledge from 0 to 4 ,0=not at all aware;4=very well aware) |
| 64 | Are you aware of the recommendations to be followed in case of pregnant women before travel to a malaria endemic region? (rate your knowledge from 0 to 4 ,0=not at all aware;4=very well aware) |
| 65 | Are you aware about the contraindications and adverse effects of common antimalarial drugs? (rate your knowledge from 0 to 4 ,0=not at all aware;4=very well aware) |
| 66 | Do you know the definition of traveller’s diarrhoea? (0=no;1= not sure; 2=yes) |
| 67 | Will you be able to identify travellers who are at risk of traveller’s diarrhoea? (rate your ability from 0 to 4, 0=very unlikely, 4=very likely) |
| 68 | Will you be able to educate a patient regarding the behaviour modification for preventing traveller’s diarrhoea? (rate your ability from 0 to 4, 0=very unlikely, 4=very likely) |
| 69 | Will you be able to prescribe anti-microbial pharmacological prophylaxis against traveller’s diarrhoea? (rate your ability from 0 to 4, 0=very unlikely, 4=very likely) |
| 70 | Are you aware of the common mental disorders associated with travel? (rate your knowledge from 0 to 4 ,0=not at al aware 4=very well aware) |
| 71 | Are you aware of the concept called culture shock? (rate your knowledge from 0 to 4 ,0=not at al aware 4=very well aware) |
| 72 | Are you aware of the special issues to be addressed in case of HIV infected traveller? (rate your knowledge from 0 to 4 ,0=not at al aware 4=very well aware) |
| 73 | Are you confident enough to give pre-travel counselling to a traveller with HIV/AIDS according to their CD4 count/ viral load? (rate from 0 to 4, 0=not confident at all;4=very much confident) |
| 74 | Are you aware of the equipment and supplies required for starting travel clinic? (rate your knowledge from 0 to 4 ,0=not at al aware 4=very well aware) |
| 75 | Are you aware of the information resources for a clinician required for conducting a travel clinic? (rate your knowledge from 0 to 4 ,0=not at al aware 4=very well aware) |
| 76 | Are you aware of the information to be included in an immunisation record? (rate your knowledge from 0 to 4 ,0=not at al aware 4=very well aware) |
| 77 | Are you aware of the common problems encountered in travel clinic practices? (rate your knowledge from 0 to 4 ,0=not at al aware 4=very well aware) |
| 78 | Will you be able to advice the basic risk mitigation measures (to minimize the risk of getting disease) before long distance travel in the context of Covid-19? (rate your ability from 0 to 4, 0=very unlikely, 4=very likely) |
| 79 | Are you aware of the conditions where an incoming traveller to your country/ locality should be advised quarantine in the context of Covid-19? (rate your knowledge from 0 to 4 ,0=not at al aware 4=very well aware) |
| 80 | Are you aware of the concept called ‘immunity passport’ in the context of COVID-19 pandemic? (rate your knowledge from 0 to 4 ,0=not at all aware 4=very well aware) |
| 81 | Will you be able to tell your patient the additional items to be included in the travel medical kit in the context of COVID-19 pandemic? (rate your ability from 0 to 4, 0=very unlikely, 4=very likely) |
| 82 | Do you think that a separate Travel health insurance is required prior to travel even if the traveller possess a general health insurance (0=no;1= not sure; 2=yes) |
| 83 | Will you be able to formulate differential diagnosis based on the symptoms with which a patient present to you after travel to a particular location? (rate your ability from 0 to 4, 0=very unlikely, 4=very likely) |

| **Attitude** | |
| --- | --- |
| 84 | It is the responsibility of a practicing physician to provide information on travel medicine to a traveller (Strongly disagree=0, disagree=1, neither agree or disagree=2, agree=3 strongly agree=4) |
| 85 | The basic knowledge on travel medicine is a part of competence of a practicing physician in the modern world scenario (Strongly disagree=0, disagree=1, neither agree or disagree=2, agree=3 strongly agree=4) |
| 86 | Body of knowledge of travel medicine differs from general medicine, infectious disease and tropical medicine? (0=no;1= not sure; 2=yes) |
| 87 | There should be a training program on travel medicine for medicine and infectious diseases at your health centre (Strongly disagree=0, disagree=1, neither agree or disagree=2, agree=3 strongly agree=4) |
| 88 | Are you interested in participating in a short course on travel medicine at your health centre? (0=no;1= not sure; 2=yes) |
| 89 | Knowledge on travel vaccines is important for a practising physician (Strongly disagree=0, disagree=1, neither agree or disagree=2, agree=3 strongly agree=4) |
| 90 | Knowledge on health issues related to high altitude is important for a practising physician (Strongly disagree=0, disagree=1, neither agree or disagree=2, agree=3 strongly agree=4) |
| 91 | Knowledge on health issues related to extremes of climate and its management is important for a practising physician (Strongly disagree=0, disagree=1, neither agree or disagree=2, agree=3 strongly agree=4) |
| 92 | Knowledge on health issues related to scuba diving is important for a practising physician (Strongly disagree=0, disagree=1, neither agree or disagree=2, agree=3 strongly agree=4) |
| 93 | Knowledge health risks associated with stings/bites and envenomation is important for a practising physician (Strongly disagree=0, disagree=1, neither agree or disagree=2, agree=3 strongly agree=4) |
| 94 | Knowledge of food and drinking water borne health risks is important for a practising physician (Strongly disagree=0, disagree=1, neither agree or disagree=2, agree=3 strongly agree=4) |
| 95 | Knowledge of vector borne disease prevention and malaria chemoprophylaxis is important for a practising physician (Strongly disagree=0, disagree=1, neither agree or disagree=2, agree=3 strongly agree=4) |
| 96 | Knowledge of risk of travellers’ diarrhoea and its prophylaxis is important for a practising physician (Strongly disagree=0, disagree=1, neither agree or disagree=2, agree=3 strongly agree=4) |
| 97 | Addressing mental health issues post travel is important in post travel consultation (Strongly disagree=0, disagree=1, neither agree or disagree=2, agree=3 strongly agree=4) |
| **Practice** | |
| 98 | How often have you done pre-travel assessment of a traveller before travel in the last 2 years? (1=Never, 2=Rarely, 3=Sometimes, 4=Often) |
| 99 | How often have you given pre-travel advice for your patient planning specifically for air travel in the last 2 years? (1=Never, 2=Rarely, 3=Sometimes, 4=Often) |
| 100 | How often have you advised travel vaccines in the last 2 years? (1=Never, 2=Rarely, 3=Sometimes, 4=Often) |
| 101 | How often have you given pre-travel advice to a person planning for a trip to high altitude in the last 2 years? (1=Never, 2=Rarely, 3=Sometimes, 4=Often) |
| 102 | How often have you given pre-travel advice to a patient expected to meet with extremes of climate in the last 2 years? (1=Never, 2=Rarely, 3=Sometimes, 4=Often) |
| 103 | How often have you given health advice to a person planning for scuba diving in the last 2 years? (1=Never, 2=Rarely, 3=Sometimes, 4=Often) |
| 104 | How often have you given pre-travel advice to a person planning for attending mass gathering in the last 2 years? (1=Never, 2=Rarely, 3=Sometimes, 4=Often) |
| 105 | How often have you advised malaria chemoprophylaxis to a person planning to travel to a malaria endemic region the last 2 years? (1=Never, 2=Rarely, 3=Sometimes, 4=Often) |
| 106 | How often have you advised prophylaxis for travellers’ diarrhoea in the last 2 years? (1=Never, 2=Rarely, 3=Sometimes, 4=Often) |
|  | © Arvind Kumar et al, All India Institute of Medical Sciences, New Delhi |
